# Comparative evaluation of three total full-face masks for delivering Non-Invasive Positive Pressure Ventilation (NPPV): a bench study

**DOI:** 10.1186/s12890-023-02489-2

**Published:** 2023-05-31

**Authors:** Giorgia Spinazzola, Giuliano Ferrone, Roberta Costa, Marco Piastra, Gianmarco Maresca, Marco Rossi, Massimo Antonelli, Giorgio Conti

**Affiliations:** 1 Department of Emergency, Intensive Care Medicine and Anesthesia, Fondazione Policlinico Universitario A. Gemelli IRCCS, Largo Francesco Vito N 8, 00168 Rome, Italy; 2 Istituto Di Anestesiologia E Rianimazione, Università Cattolica del Sacro Cuore, Largo Francesco Vito 8, Rome, Italy

**Keywords:** Non-invasive positive pressure ventilation, Respiratory failure, Patient-ventilator interaction

## Abstract

Historically, the oro-nasal mask has been the preferred interface to deliver Non-Invasive Positive Pressure Ventilation (NPPV) in critically ill patients. To overcome the problems related to air leaks and discomfort, Total Full-face masks have been designed. No study has comparatively evaluated the performance of the total Full-face masks available.The aim of this bench study was to evaluate the influence of three largely diffuse models of total Full -face masks on patient-ventilator synchrony and performance during pressure support ventilation. NPPV was applied to a mannequin, connected to an active test lung through three largely diffuse Full-face masks: Dimar Full-face mask (DFFM), Performax Full-face mask (RFFM) and Pulmodyne Full-face mask (PFFM).The performance analysis showed that the ΔPtrigger was significantly lower with PFFM (*p* < 0.05) at 20 breaths/min (RRsim) at both pressure support (iPS) levels applied, while, at RRsim 30, DFFM had the longest ΔPtrigger compared to the other 2 total full face masks (*p* < 0.05). At all ventilator settings, the PTP200 was significantly shorter with DFFM than with the other two total full-face masks (*p* < 0.05). In terms of PTP500 ideal index (%), we did not observe significant differences between the interfaces tested.

The PFFM demonstrated the best performance and synchrony at low respiratory rates, but when the respiratory rate increased, no difference between all tested total full-face masks was reported.

## Introduction

Non-invasive Positive Pressure Ventilation (NPPV) is a technique of ventilatory assistance used as a first-line treatment for acute respiratory failure of different origins [1–7].

The main advantages of NPPV are: 1) avoiding the side effects and complications related to endotracheal intubation [8], 2) improving patient comfort and 3) preserving airway defense mechanisms. An important issue for NPPV success is the choice of an appropriate interface.

Currently, different types of interfaces for NPPV are available, including nasal masks, oro-nasal mask, total Full-face masks and helmets [9, 10]. The oro-nasal mask has historically been the preferred interface to deliver NPPV in critically ill patients [11, 12]. Despite an appropriate clinical indication, a relatively high percentage of patients fail NPPV delivered by oro-nasal mask due to air leaks, discomfort and claustrophobia [13, 14]. To overcome these problems, total Full-face masks have been designed in order to increase patient comfort by maintaining a tight seal around the face. In fact, these interfaces create an air seal trough a silicon gasket around the whole perimeter of the face [15], thus eliminating the discomfort generated by the application of pressure over the nasal bridge.

To our best knowledge, while several studies analyzed the performance of different models of helmets for NPPV [16, 17], no study has comparatively evaluated the performance of the total Full-face masks available.

The aim of this bench study was to evaluate the influence of three largely diffuse models of total full-face masks connected through a standard Y-piece to a double circuit on patient-ventilator synchrony and performance during pressure support ventilation with different breathing frequencies, times of pressurization, and cycling-off flow thresholds.

## Material and methods

The study was performed at the Respiratory Mechanics Laboratory (Ventil@b) of the Fondazione Policlinico Universitario A. Gemelli IRCCS, Università Cattolica del Sacro Cuore in Rome, Italy.

### Bench study

NPPV was applied to a mannequin (Laerdal Medical AS, Stavanger, Norway), connected to an active test lung (ASL 5000; Ingmar Medical, Pittsburgh, PA, USA), through three largely diffuse total Full-face masks connected through a standard double circuit with a Y-piece: Dimar total Full-face mask (DFFM) (Mirandola, Italy), Performax total Full-face mask (RFFM) (Philips, Respironics, Murrysville, PA, USA) and Pulmodyne total Full-face mask (PFFM) (BiTrac MaxShield, Indianapolis, IN, USA). During the study, we tested the small adult size of all interfaces.

The interfaces tested are characterized by a total facial perimeter cushion made with soft silicone and an adjustable nuchal fixing system. The three models of interfaces tested presented slightly different inner volume: DFFM and PFFM have an inner volume of 660 ml, while RFFM has an inner volume of 700 ml.

Total Full-face mask NPPV was delivered, at simulator respiratory rates (RRsim) of 20 and 30/min, with a mechanical ventilator with NIV module (Puritan Bennet 840; Covidien Health Care, Mansfield, MA) set at two different inspiratory pressure support levels (iPS) 10 and 15 cmH_2_O, with Positive End-Expiratory Pressure (PEEP) 8 cmH_2_O. During the bench test, the ventilator was set to optimize patient-ventilator interaction by choosing the fastest pressurization ramp (100%), two values of expiratory cycling off (25% of peak inspiratory flow, slow setting and 50% of peak inspiratory flow, fast setting), checking for the absence of premature mechanical inspiratory termination, and a flow trigger set at the lowest value thus avoiding autotrigger phenomena.

Each test condition lasted for 20 min, recording continuously the last 5 min.

The ASL 5000 is a digitally controlled real-time breathing simulator, that allows the creation of various types of breaths as during spontaneous ventilation. A broad range of flow (from 1 to 180 l/min, with a rise time < 50 ms), tidal volume (from 2 to 2500 ml), respiratory rate (from 0 to 150 breaths/min), resistance (between 3 and 500 cmH_2_O/l/s), compliance (from 0.5 to 250 ml/cmH_2_O) and inspiratory muscle effort (from 0 to 100 cmH_2_O) can be reproduced.

For the purposes of this study, the ASL 5000 active test lung system was set using a single-compartment model, an active inspiration simulated by a semi-sinusoidal pressure waveform (Rise Time 15%, Pause 0% and Release Hold 25%). The lung simulator was set to mimic an adult patient of 70 kg Body weigh with mild restrictive acute respiratory failure, breathing with an inspiratory effort (Pmus) of 8 cmH_2_O, a respiratory system compliance of 40 ml/cmH_2_O and a respiratory resistance of 4 cmH_2_O/L/sec.

### Measurements

The airflow delivered by the ventilator to the total face mask (V’) during the inspiratory phase was measured with a pneumotachograph (Fleisch n.2; Metabo, Epalinges, Switzerland) positioned at the Y-connection of the ventilator circuit. The airway pressure (Paw) of the inspiratory limb of the circuit was measured by a pressure transducer with a differential pressure of ± 100 cmH_2_O (Digima Clic-1; KleisTEK, ICU-Lab System, Italy), placed distally to the pneumotachograph. All the signals were acquired, amplified, filtered, digitized at 100 Hz, recorded on a dedicated personal computer, and analyzed with a specific software (ICU lab 2.3; KleisTEK Advanced Electronic System, Italy and Analysis Plus; Novametrix Medical System, Wallingford, CT, USA). Ventilator inspiratory and expiratory time (Timec and Temec, respectively), and ventilator rate of cycling (RRmec) were all determined on the Flow (V’) tracing. The inspiratory duty cycle (Ti/Ttot) was calculated as the ratio between Timec and the sum of Timec and Temec (Ttot). Airflow (V’) and tidal volume (Vt) delivered to the simulator, airway opening pressure (Paw), and Pmus were displayed online on the computer screen. The signals obtained with the ASL were transmitted to a PC host via 10/100 MBit Ethernet, sampled, and processed in real time. by means of a specific software (Lab View; Ingmar Medical, Pittsburgh, PA, USA). The amount of tidal volume delivered to the simulator during its active inspiration (i.e., while Pmus is negative) (i.e., the simulator tidal volume, VTsim) was calculated as the volume generated from the onset of Pmus negative deflection to its return to baseline [17–20].

Interfaces performance was evaluated by measuring the following parameters [17–20]:

- Trigger pressure drop (∆Ptrigger), defined as the pressure drop generated in triggering the ventilator.
- Inspiratory pressure–time product (PTPt), defined as the area under Paw between the onset of inspiratory effort and of mechanical assistance.
- Pressure time product 200 ms from the onset of the ventilator pressurization (PTP200), as an index of pure pressurization performance.
- Pressure–time product at 300 and 500 ms (PTP 300 and PTP 500), variables defining the speediness of pressurization and the ventilator capacity to maintain the set pressure.
- PTP 500 ideal index, expressed the percentage of ideal PTP, which is unattainable because it would imply a trigger pressure drop and an instantaneous pressurization of the ventilator.

Patient-ventilator interaction was evaluated by determining [21–23]:

- Pressurization time (Time_press_), defined as the time necessary to achieve the preset level of pressure support.
- Inspiratory trigger delay (Delay_trinsp_), calculated as the time lag between the onset of Pmus negative swing and the start of the ventilator support (i.e., Paw positive deflection).
- Expiratory trigger delay (Delay_trexp_), as assessed as the delay between the offset of the inspiratory effort and the offset of the mechanical insufflation.
- Time of synchrony (Time_sync_), defined as the time during which inspiratory muscle effort and Paw are in phase.
- Simulator VT/mechanical VT, intended as the percentage of Vt delivered during inspiratory muscle effort negative deflection.
- The time during which simulator respiratory effort and ventilator assistance were synchronous, indexed to the simulator inspiratory time (Timesync/Tineu) was also computed.
- The number of wasted (ineffective) efforts, defined as ineffective inspiratory efforts, not assisted by the ventilator.
- Numbers of Autotrigger phenomena, namely a mechanical insufflation in absence of inspiratory effort.

### Statistical analysis

Continuous data were expressed as mean and standard deviation (SD). Categorical data were presented as numbers and percentage in brackets. All variables were compared with each interface used. Comparisons were made by Student's t- test and Chi test, as appropriate. The analysis of variance (ANOVA) for repeated measures was used to detect significant differences between the different experimental conditions. When significant differences were detected, a post hoc analysis was performed using the Bonferroni test; *p* values < 0.05 were considered statistically significant. Statistical analysis was performed using MEDcalc version 18.6.

## Results

**Fig. 1:**
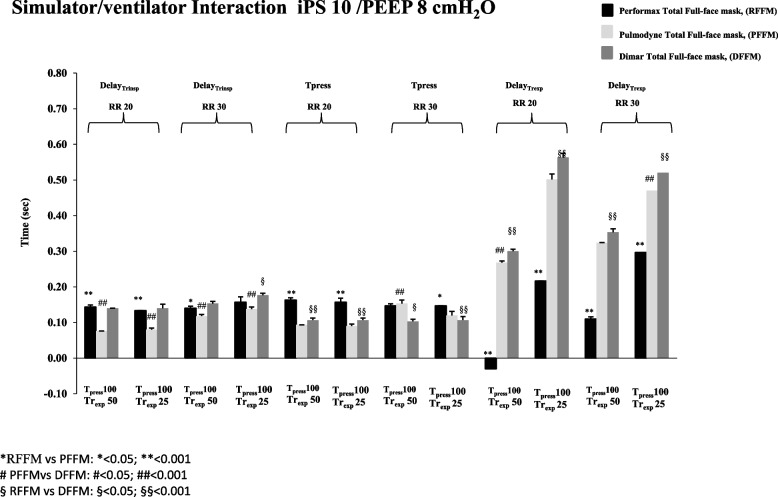
Inspiratory trigger delay (Delaytr_insp_), Expiratory Trigger delay (Dealytr_exp_) and Pressurization Time (T_press_) with Performax Total full face mask (RFFM) (black column), Pulmodyne Total full face mask (PFFM)(light gray column), and Dimar Total full face mask (DFFM) (dark grey column) at 20 and 30 breaths/min, with inspiratory pressure support (iPS) of 10 cmH2O and Positive End Expiratory Pressure (PEEP) of 8 cmH2O

The analysis of the patient-ventilator interaction showed that, independently from the ventilator setting applied, at 20 and 30 breaths/min and PS 10 cmH_2_O, no significant difference was observed between the RFFM and the DFFM in terms of Delay_trinsp_, while the PFFM showed significantly reduced values of this variable (*p* < 0.01) (Fig. 1).

The Pressurization Time (Time_press_) showed that, at 20 breaths/min and iPS 10, PFFM presented a significantly faster Time_press_ compared to the other 2 masks (*p* < 0.05), while at 30 breaths/min with the same iPS values DFFM showed the shorter value of Time_press_ compared to the other two total face tested. In terms of Delay_trexp_, at the study conditions above mentioned, RFFM presented the shorter Delay_trexp_ compared to the other two Full-face masks (*p* < 0.01), while with the fast setting and at 20 breaths/min this Full- face mask showed a premature termination of the mechanical insufflation (Fig. 1).

**Fig. 2:**
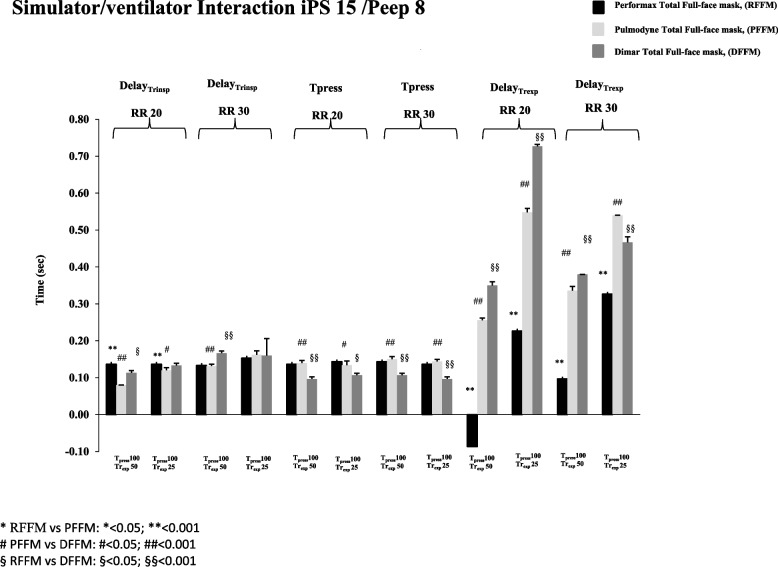
Inspiratory trigger delay (Delaytr_insp_), Expiratory Trigger delay (Dealytr_exp_) and Pressurization Time (T_press_) with Performax Total full face mask (RFFM) (black column), Pulmodyne Total full face mask (PFFM)(light gray column), and Dimar Total full face mask (DFFM) (dark grey column) at 20 and 30 breaths/min, with inspiratory pressure support (iPS) of 15 cmH2O and Positive End Expiratory Pressure (PEEP) of 8 cmH2O

At iPS 15 cmH_2_O and 20 breaths/min, Delay_trinsp_ was shorter with PFFM compared both to DFFM and RFFM(p < 0.05), while at 30 breaths/min with the fast-setting, DFFM showed a significant prolongation of Delay_trinsp_ compared to the other two masks (*p* < 0.01) (Fig. 2) while with the slow setting no differences were observed between the three Full-face masks tested.

The Time_press_ analysis showed that, at both RRsim tested and independently from the setting applied, DFFM had a shorter Time_press_ compared to the other two Full-face masks tested (*p* < 0.05), while the Delay_trexp_ was significantly longer with this mask in almost all the study conditions (*p* < 0.01) (Fig. 2).

**Fig. 3:**
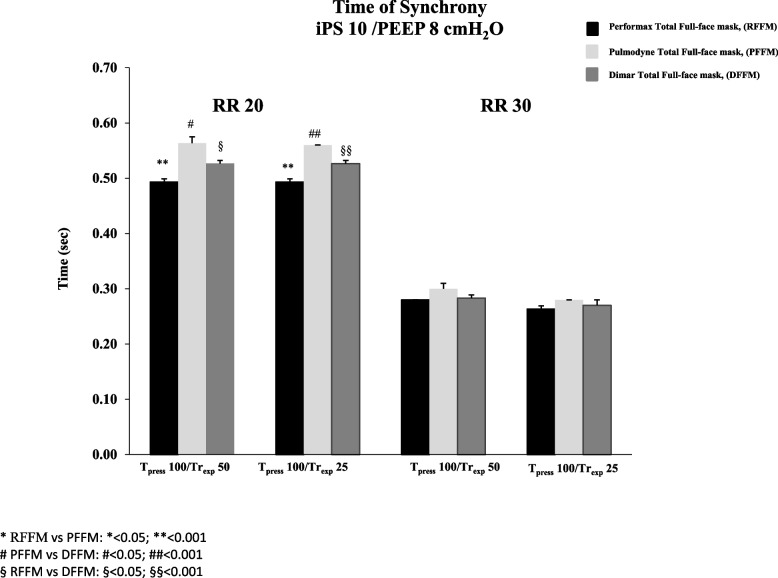
Time of synchrony with Performax Total full face mask (RFFM) (black column), Pulmodyne Total full face mask (PFFM)(light gray column), and Dimar Total full face mask (DFFM) (dark grey column) at two respiratory rates (RR 20 and 30 breaths/min), with inspiratory pressure support (iPS) of 10 cmH2O and Positive End Expiratory Pressure (PEEP) of 8 cmH2O

**Fig. 4:**
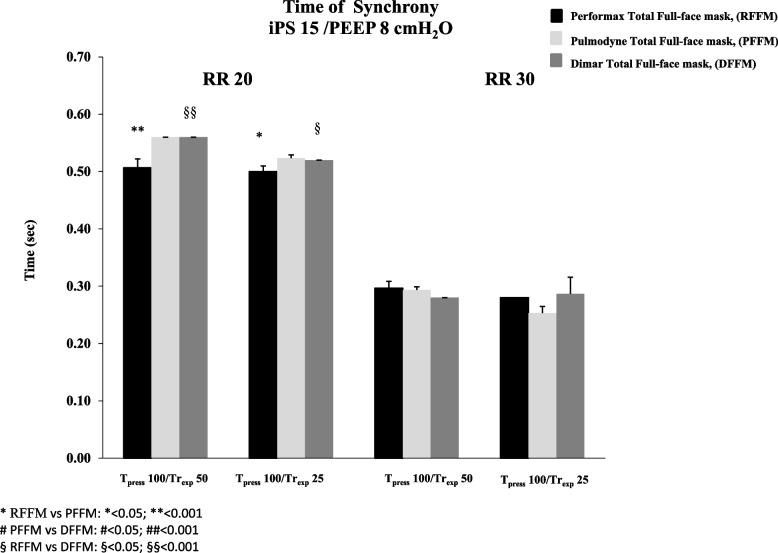
Time of synchrony with Performax Total full face mask (RFFM) (black column), Pulmodyne Total full face mask (PFFM) (light gray column), andDimar Total full face mask (DFFM) (dark grey column) at two respiratory rates (RR 20 and 30 breaths/min), with inspiratory pressure support (iPS) of 15 cmH2O and Positive End Expiratory Pressure (PEEP) of 8 cmH2O

The Time_sync_ analysis showed that, at both iPS levels tested, at 30 breaths/min no significant difference was found between the three interfaces, while, at 20 breaths/min and at both iPS levels applied, a significant prolongation of the Time_sync_ with PFFM and DFFM compared to the RFFM (*p* < 0.05) was observed, even though PFFM showed, only at RR 20, the longer Time_sync_ compared to the other two masks (*p* < 0.05) (Figs. 3 and 4).

At all ventilator settings, the RFFM delivered the lower value of mechanical VT in comparison with PFFM and DFFM (*p* < 0.05), while no difference was reported in terms of mechanical VT between PFFM and DFFM. Similar results were obtained in terms of simulator VT/mechanical VT. In fact, during all bench study conditions, DFFM showed the lower value of simulator VT/mechanical VT compared to the other full-face masks.

The three total Full-face masks tested did not present significant differences in terms of Timesynch/Tineu during the whole bench study.

The performance analysis showed that, independently from the setting, the ∆Ptrigger was significantly lower with PFFM (*p* < 0.05) at 20 RRsim at both iPS levels applied, while, at RRsim 30, DFFM had the longest ∆Ptrigger compared to the other 2 total full-face masks (*p* < 0.05) (Tables 1 and 2).

**Table 1:** Performance of the interfaces during NPPV at 20 and 30 RRsim, iPS 10 and PEEP 8 cmH2O

	***RFFM***	***PFFM***	***DFFM***		***RFFM***	***PFFM***	***DFFM***	
	***RR 20 Timepress100%/Trexp50%***	***RR 20 Timepress100%/Trexp50%***	***RR20 Timepress100%/Trexp50%***	***P***	***RR 20 Timepress100%/Trexp25%***	***RR 20 Timepress100%/Trexp25%***	***RR20 Timepress100%/Trexp25%***	***P***
**ΔPtrigger (cmH**_**2**_**O)**	1.01 ± 0.020.02	0.40 ± 0.03	1.08 ± 0.02	0.05	0.99 ± 0.04	0.45 ± 0.05	1.08 ± 0.02	0.05
**PTPt (cmH**_**2**_**O/s)**	0.06 ± 0.01	0.02 ± 0.01	0.06 ± 0.01	0.01	0.06 ± 0.01	0.02 ± 0.01	0.07 ± 0.01	0.05
**PTP200 (cmH**_**2**_**O/s)**	1.37 ± 0.02	1.05 ± 0.02	0.4 ± 0.08	0.05	1.21 ± 0.02	0.92 ± 0.03	0.38 ± 0.05	0.05
**PTP300 (cmH**_**2**_**O/s)**	1.19 ± 0.05	1.31 ± 0.05	1.18 ± 0.12	0.05	1.06 ± 0.02	1.13 ± 0.06	1.23 ± 0.03	0.05
**PTP500 ideal index (%)**	61	74	67	0.8	60	69	67	0.8
	***RR 30*** ***Timepress100%/Trexp50%***	***RR 30*** ***Timepress100%/Trexp50%***	***RR30 Timepress100%/Trexp50%***	***P***	***RR 30*** ***Timepress100%/Trexp25%***	***RR 30*** ***Timepress100%/Trexp25%***	***RR30 Timepress100%/Trexp25%***	***P***
**ΔPtrigger (cmH**_**2**_**O)**	1.26 ± 0.06	1.42 ± 0.03	2.09 ± 0.04	0.05	1.71 ± 0.03	1.71 ± 0.01	2.32 ± 0.02	0.05
**PTPt (cmH**_**2**_**O/s)**	0.07 ± 0.01	0.09 ± 0.01	0.16 ± 0.01	0.05	0.13 ± 0.01	0.13 ± 0.01	0.21 ± 0.01	0.01
**PTP200 (cmH**_**2**_**O/s)**	1.38 ± 0.03	0.89 ± 0.06	0.30 ± 0.01	0.05	1.32 ± 0.02	0.76 ± 0.01	0.17 ± 0.03	0.05
**PTP300 (cmH**_**2**_**O/s)**	1.18 ± 0.02	0.89 ± 0.02	1.17 ± 0.01	0.05	0.85 ± 0.01	0.71 ± 0.01	1.02 ± 0.06	0.05
**PTP500 ideal index (%)**	62	60	65	0.26	51	56	62	0.26

*ΔPtrigger* trigger pressure drop, *PTPt* Pressure Time Product during the triggering phase, *PTP200* and *PTP300* Pressure Time Product during the initial 200 and 300 from the onset of the ventilator pressurization expressed as the absolute value, *PTP500 ideal index* Pressure Time Product during the initial 500 ms from the onset of the simulated effort, expressed as the percentage of the area of ideal pressurization, with different ventilator settings (see text). *DFFM* Dimar Full-face mask*, RFFM* Performax Full-face mask and *PFFM* Pulmodyne Full-face mask, *RR* Respiratory rates, *iPS* inspiratory pressure support, *NPPV* Non-Invasive Pressure Support Ventilation

**Table 2:** Performance of the interfaces during NIV at 20 and 30 RRsim, iPS 15 and PEEP 8 cmH2O

	***RFFM***	***PFFM***	***DFFM***		***RFFM***	***PFFM***	***DFFM***	
	***RR 20 Timepress100%/Trexp50%***	***RR 20 Timepress100%/Trexp50%***	***RR20 Timepress100%/Trexp50%***	***P***	***RR 20 Timepress100%/Trexp25%***	***RR 20 Timepress100%/Trexp25%***	***RR20 Timepress100%/Trexp25%***	***P***
**ΔPtrigger (cmH**_**2**_**O)**	0.99 ± 0.02	0.48 ± 0.06	0.82 ± 0.07	0.05	0.98 ± 0.04	1.00 ± 0.06	1.09 ± 0.08	0.3
**PTPt (cmH**_**2**_**O/s)**	0.06 ± 0.01	0.02 ± 0.01	0.04 ± 0.01	0.03	0.05 ± 0.01	0.07 ± 0.01	0.06 ± 0.01	0.13
**PTP200 (cmH**_**2**_**O/s)**	1.37 ± 0.02	1.05 ± 0.02	0.40 ± 0.08	0.05	1.21 ± 0.02	0.92 ± 0.03	0.38 ± 0.05	0.05
**PTP300 (cmH**_**2**_**O/s)**	1.19 ± 0.05	1.31 ± 0.05	1.18 ± 0.12	0.05	1.06 ± 0.02	1.13 ± 0.06	1.23 ± 0.03	0.05
**PTP500 ideal index (%)**	64	71	63	0.58	60	65	67	0.58
	***RR 30*** ***Timepress100%/Trexp50%***	***RR 30*** ***Timepress100%/Trexp50%***	***RR30 Timepress100%/Trexp50%***	***P***	***RR 30*** ***Timepress100%/Trexp25%***	***RR 30*** ***Timepress100%/Trexp25%***	***RR30 Timepress100%/Trexp25%***	***P***
**ΔPtrigger (cmH**_**2**_**O)**	1.33 ± 0.03	1.68 ± 0.08	2.17 ± 0.05	0.05	1.44 ± 0.09	2.03 ± 0.03	2.17 ± 0.19	0.05
**PTPt (cmH**_**2**_**O/s)**	0.08 ± 0.01	0.12 ± 0.01	0.18 ± 0.01	0.05	0.11 ± 0.01	0.21 ± 0.02	0.19 ± 002	0.05
**PTP200 (cmH**_**2**_**O/s)**	2.01 ± 0.03	1.45 ± 0.03	0.31 ± 0.09	0.05	2.06 ± 0.04	1.19 ± 0.04	0.24 ± 0.20	0.05
**PTP300 (cmH**_**2**_**O/s)**	1.80 ± 0.08	1.35 ± 0.06	1.57 ± 0.10	0.60	1.61 ± 0.10	0.92 ± 0.04	1.38 ± 0.25	0.05
**PTP500 ideal index (%)**	63	60	63	0.34	55	53	62	0.34

*ΔPtrigger* trigger pressure drop, *PTPt* Pressure Time Product during the triggering phase, *PTP200* and *PTP300* Pressure Time Product during the initial 200 and 300 from the onset of the ventilator pressurization expressed as the absolute value, *PTP500 ideal index* Pressure Time Product during the initial 500 ms from the onset of the simulated effort, expressed as the percentage of the area of ideal pressurization, with different ventilator settings (see text). *DFFM* Dimar Full-face mask*, RFFM* Performax Full-face mask and *PFFM* Pulmodyne Full-face mask, *RR* Respiratory rates, *iPS* inspiratory pressure support, *NPPV* Non-Invasive Pressure Support Ventilation

The PTP_t_ analysis showed that, at RRsim 20, PFFM had a smaller PTP_t_ than the other 2 total full-face masks at iPS 10, independently from the setting used, and at iPS 15 only during the fast setting. Finally, at RRsim 30 in all conditions tested, RFFM showed a shorter PTP_t_ compared to the other two total Full-face masks (Tables 1 and 2).

At all ventilator settings, the PTP200 value was significantly shorter with DFFM than with the other two total full-face masks (*p* < 0.05). In terms of PTP 300, we observed that, at RRsim 20 and fast setting, PFFM presented the significantly longest value compared to the others two masks at both iPS and PEEP tested, while at RRsim 20 with slow setting the DFFM presented the significantly longest value (Tables 1 and 2).

At RRsim 30, in all conditions tested, the PFFM presented the shorter value of PTP 300 compared to the other total full-face masks (*p* < 0.05).

Finally, the PTP 500 indexed did not show significant differences between the three interfaces tested (Tables 1 and 2).

## Discussion

The results of this bench study show that the physical characteristics and the design of the total Full-face mask may influence patient–ventilator interaction during NPPV.

NPPV delivered through a total full-face mask can be a valid alternative to the oro-nasal mask for the treatment of patients with respiratory failure [24].

Several studies described the conditions associated to NPPV failure with the use of oro-nasal mask [25–28] as intolerance, attributed to mask discomfort or poor fit, excessively tightened straps, excessive air leaks, patient-ventilator asynchrony, or claustrophobia. The total Full-face mask has been designed to overcome the drawbacks related to the oro-nasal mask use [29].

Several studies have been conducted to explore the efficacy of total Full-face masks in comparison with the oro-nasal mask, both in terms of comfort and clinical outcomes, expressed as rate of endotracheal intubation, clinical evolution and gas exchange [29, 30]. In a recent study, Sadeghi and coll [31] compared the total full-face mask and the oro-nasal mask in terms of effectiveness and comfort in patients with acute respiratory failure treated with NPPV. During this study, the authors enrolled 48 patients with acute respiratory failure treated with NPPV (24 patients who applied the total full-face mask and 24 patients who applied the oro-nasal mask). The authors demonstrated the non-inferiority of total full-face mask in comparison with the oro-nasal mask in terms of clinical outcome and reported a lower score for cheeks pain in patients receiving NPPV via full face mask.

In the last years, several bench studies [16–18, 23] were conducted to evaluate the impact of interface physical characteristics and the role of the optimal choice of the circuit on interface performance and patient-ventilator interaction.

Moreover, in a recent study, Ferrone et al. demonstrated that the presence of 2 different connectors for inflow and outflow gases improves the interaction and performance of the only total full-face mask equipped with this kind of circuit present in the market [32]. However, the aim of the present study was to perform a head-to-head comparison of three total full-face masks largely diffuse in the market and connected through a standard Y-piece to a double circuit.

To date, although different models of total face masks are available in Europe for clinical use, to our best knowledge, no study has evaluated the impact of their different physical characteristics on patient–ventilator interaction during NPPV.

In our study, despite a better patient-ventilator interaction with the PFFM in terms of Delay_trinsp_ and Time_press_ during iPS 10 and PEEP 8 cmH2O, with increased values of iPS also DFFM showed a better interaction respect to RFFM. The Time of Synchrony analysis showed that at high RR (30 breath/min) no significant differences were found between the three interfaces, while, at RR 20, PFFM showed a longer Time_sync_ compared to the other two total full-face masks. However, it is worth to underline that the three interfaces were all able, at lower RR, to guarantee a Time_sync_ above 400 ms, and thus to assist more than 50% of the duration of the simulator breath.

Concerning the performance, our results showed that PFFM presented the better performance at low RRsim, as demonstrated by the lower ∆Ptrigger and PTPt, while increasing the RRsim during iPS 10 the PFFM and RFFM showed a similar performance. At iPS 15 and high RRsim, the RFFM presented the better performance compared to the other two total full-face masks. Furthermore, RFFM showed the longest PTP 200 compared to the other two total full-face masks.

During this bench study, the DFFM presented a reduced performance increasing iPS and RRsim respect to the other interfaces tested, showing the longest values of ∆Ptrigger and PTPt.

These results can be partially explained by considering the different shape and material of the DFFM compared to the other two masks. In particular, the softer material of the flange, created to increase patient comfort, causes an initial pressure dissipation during the mechanical pressurization, with a consequently longer Delay_trinsp,_ ∆Ptrigger and PTP_t,_ while, once the interface is well pressurized, the time to reach the preset level of iPS is faster with DFFM than with the other 2 masks [33].

This study has an important limitation, being a bench physiological study, and our results need confirmation in a clinical study performed in critically ill patients. In this bench study, we did not test a vented circuit configuration, as our aim was to evaluate patient ventilator interaction and performance of total full-face masks in an ICU settings, where this specific kind of circuit is widely used, while vented circuits are generally used outside the ICU in chronic respiratory failure settings and/or in stepdown units. Moreover, in our study, we did not perform a comparison between these total full-face masks with a standard circuit with the only model present on the market equipped with embedded double inflow and outflow circuits, that is probably more performant [32] but is very recent and not largely diffuse.

## Conclusion

In conclusion, the results of this comparative bench study suggest that the physical characteristics and different design of the total Full-face masks can influence patient–ventilator interaction and performance. Moreover, different type of total face mask may perform diversely in delivering NPPV with various ventilator settings and breathing frequencies. The PFFM demonstrated a better performance and assistance at low respiratory rate compared to the others full-face masks tested, but when the respiratory rate increased all full-face masks showed a similar behaviour. This aspect should be considered when choosing a total Full-face mask for clinical use.

## Data Availability

The data generated or analyzed during current study are available from the corresponding author on reasonable request.
